# The Long-Term Impact of Maternal Anxiety and Depression Postpartum and in Early Childhood on Child and Paternal Mental Health at 11–12 Years Follow-Up

**DOI:** 10.3389/fpsyt.2020.562237

**Published:** 2020-09-15

**Authors:** Annika L. Walker, Priya H. Peters, Susanne R. de Rooij, Jens Henrichs, Anke B. Witteveen, Corine J. M. Verhoeven, Tanja G. M. Vrijkotte, Ank de Jonge

**Affiliations:** ^1^ Department of Midwifery Science, Amsterdam UMC, Vrije Universiteit Amsterdam, AVAG–Amsterdam Public Health Research Institute, Amsterdam, Netherlands; ^2^ Department of Public and Occupational Health, Amsterdam UMC, University of Amsterdam, Amsterdam Public Health Research Institute, Amsterdam, Netherlands; ^3^ Centre for Urban Mental Health, University of Amsterdam, Amsterdam, Netherlands; ^4^ Department of Clinical Epidemiology, Amsterdam UMC, University of Amsterdam, Biostatistics and Bioinformatics, Amsterdam Public Health Research Institute, Amsterdam, Netherlands; ^5^ Division of Midwifery, School of Health Sciences, University of Nottingham, Nottingham, United Kingdom

**Keywords:** anxiety, depression, postpartum, family mental health, fathers, socio-emotional development, early childhood, adolescence

## Abstract

**Background:**

Postpartum maternal anxiety and depression can affect child development and family functioning. However, the long-term impact of postpartum maternal anxiety and depression on child and paternal mental health is understudied. The present large-scale prospective cohort study is one of the first to investigate whether maternal anxiety and depressive symptoms postpartum and at child age 5–6 years separately and synergistically increase paternal anxiety and depressive symptoms and child emotional problems in early adolescence at age 11–12 years. Secondly, we investigated whether concurrent paternal anxiety and depressive symptoms at child age 11–12 years moderated the association between maternal anxiety and depressive symptoms in the postpartum period and at child age 5–6 years with child emotional problems at age 11–12 years.

**Methods:**

This study is part of the Amsterdam Born Children and Development (ABCD) cohort study, the Netherlands (*N* = 2.298). Maternal postpartum anxiety and depressive symptoms were assessed using the State-Trait Anxiety Inventory (STAI) and the Center for Epidemiologic Studies Depression Scale (CES-D) at 13 weeks postpartum. Maternal anxiety and depressive symptoms at child age 5–6 years and parental anxiety and depressive symptoms at 11–12 years were assessed using the Depression Anxiety Stress Scale (DASS-21). Child emotional problems were reported by the child and a teacher using the Strengths and Difficulties Questionnaire (SDQ). Multivariable linear regression was conducted, adjusted for demographic, perinatal/obstetric confounders, and affective symptoms of the other family members at 11–12 years.

**Results:**

Neither maternal anxiety nor depressive symptoms were related to paternal depressive symptoms at child age 11–12 years, while maternal postpartum depressive symptoms, depressive symptoms at 5–6 years and maternal anxiety at 5–6 years were positively related to paternal anxiety at 11–12 years. However, effect sizes were small. Only maternal postpartum depression was positively but weakly associated with more child emotional problems at 11–12 years. Although paternal concurrent affective symptoms were positively related to more child emotional problems in early adolescence, they did not moderate the association between maternal symptoms and child emotional problems.

**Conclusions:**

Our results indicate that fathers and children seem to be affected only to a small extent by maternal postpartum anxiety or depression.

## Introduction

Maternal postpartum anxiety and depression are frequently occurring complications affecting up to 20% of young mothers and can have adverse consequences for all family members (1–4). Both maternal postpartum anxiety and depression are associated with personal suffering, psychosocial and occupational dysfunctioning, and poor family functioning and both conditions have been shown to negatively affect mother-infant interactions (2, 5–7). Maternal postpartum depression is a risk factor for both subsequent maternal mental health problems and child behavioral and emotional problems in early childhood (8, 9). Furthermore, a few studies have shown that maternal postpartum depression is also a risk factor for offspring emotional and behavioral functioning in late childhood and adolescence (10–14). However, it is not well known whether maternal postpartum anxiety also contributes to the development of children’s mental health problems later in life (15, 16).

From a developmental psychopathology perspective, a complex and dynamic interplay of psychological, social and biological mechanisms has been suggested to underlie the associations of maternal postpartum depression and anxiety with child mental health (17, 18). Next to environmental mediation, previous research indicated that genetic mediation plays, at least partly, a role in the intergenerational transmission of both depression and anxiety (19–23). Postpartum depression and anxiety can be comorbid conditions (24). However, both conditions show unique features in symptomatology. That is, low positive affect is specific for depression, while physiological hyperarousal is a unique characteristic for anxiety (25). Consequently, underlying mechanisms and ways of transmission of anxiety and depression from mother to the child may differ (23, 26–28). For example, the environmental intergenerational transmission of these two conditions may occur *via* suboptimal parenting and modeling behaviors that are specific for mothers with anxiety or depressive disorder, respectively (29–32). Furthermore, intergenerational transmission is suggested to be more specific for parental anxiety, setting children of parents with an anxiety disorder at a particularly increased risk of developing anxiety disorders themselves compared to children of parents with depressive disorders who are at an increased risk of a variety of mental problems (33). Therefore, it is important to study the long-term impact of both maternal postpartum anxiety and depression on the child (34).

Not only maternal postpartum mental health but also maternal mental health in the childhood period affects child emotional functioning. Previous studies observed that maternal anxiety and depression in (early) childhood are associated with child emotional and behavioral problems (35, 36). Both the postpartum period and the early childhood period are mentally demanding periods in a woman’s life. The postpartum period includes the transition into early motherhood while most young mothers simultaneously have to cope with extensive parenting and job demands throughout early childhood. Therefore, these periods particularly predispose women to emotional distress and anxiety. As both maternal mental health in the postpartum period and in early childhood influence child outcome, the effects of maternal anxiety or depression in these developmental periods may add up. Thus, children of mothers being highly anxious or depressed in the postpartum and early childhood period may be particularly at risk of developing childhood emotional problems. A follow-up study (*n* = 753) (37) and a recent relatively small-scale study (*n* = 474) (38) showed that maternal postpartum depression and concurrent maternal mental health problems were independently associated with more child emotional and behavioral problems at age 4 years and child internalizing problems at age 8 years, respectively. In the latter study, children of mothers with both postpartum and concurrent depression had the highest level of internalizing problems (38). These studies suggest that maternal mental health problems in both the postpartum period and in childhood affect child emotional functioning independently and synergistically. Yet, these studies analyzed cross-sectional data on child outcomes and concurrent maternal mental health in early childhood and were thus unable to address directionality of effects. Moreover, research on the long-term impact of maternal anxiety and depression in the postpartum and early childhood period on parental mental health is scarce.

According to family systems theory, family represents an organized entity wherein all family members mutually affect each other across time (39, 40). Individual mental health functioning, family relationships, and family functioning are intertwined (41). To facilitate the development and optimization of preventive interventions aimed at reducing the influence of maternal postpartum anxiety and depression on the whole family, more knowledge about the long-term impact of these maternal conditions on all family members, including the father, is needed. Although maternal postpartum anxiety and depressive symptoms have both been shown to be concurrently associated with paternal affective symptoms, their long-term impact on paternal anxiety and depressive symptoms has been understudied (42). Moreover, not only maternal anxiety and depression during the postpartum and early childhood period are associated with more child emotional problems, but also paternal mental health can affect emotional development in children (43–45). Additionally, it has been proposed that paternal mental health may moderate the association of maternal anxiety and depressive symptoms with child outcomes (9, 28). For example, paternal depression has been shown to exacerbate the effect of maternal depression on internalizing symptoms in children (46). In contrast, the absence of psychopathology in involved fathers has been suggested to buffer the negative impact of maternal depression on the child (42, 47, 48). The above illustrates that it is of great importance to increase our understanding of the long-term impact of maternal anxiety and depression in the postpartum and early childhood period on all family members. The aim of the present large-scale prospective population-based cohort study was to investigate whether maternal anxiety and depressive symptoms postpartum and at child age 5–6 years separately and synergistically increase paternal anxiety and depressive symptoms and child emotional problems in early adolescence at age 11–12 years. Secondly, we investigated whether concurrent paternal anxiety and depressive symptoms at child age 11–12 years moderated the association between maternal anxiety and depressive symptoms in the postpartum period and at child age 5–6 years with child emotional problems at age 11–12 years. We hypothesized that maternal anxiety and depressive symptoms postpartum and at child age 5–6 years synergistically increase both paternal anxiety and depressive symptoms and child emotional problems at 11–12 years. Secondly, we hypothesized that concurrent paternal anxiety and depressive symptoms at child age 11–12 years exacerbate the associations of maternal anxiety and depressive symptoms postpartum and at child age 5–6 years with child emotional problems at age 11–12 years.

## Methods

### Participants and Design

The present study is part of the Amsterdam Born Children and Development (ABCD) cohort study, an ongoing multi-ethnic population-based prospective birth cohort to identify factors in prenatal and early life explaining (differences in) child health later in life (http://www.abcd-study.nl). Details about the study design have previously been described (49).

**Figure 1:**
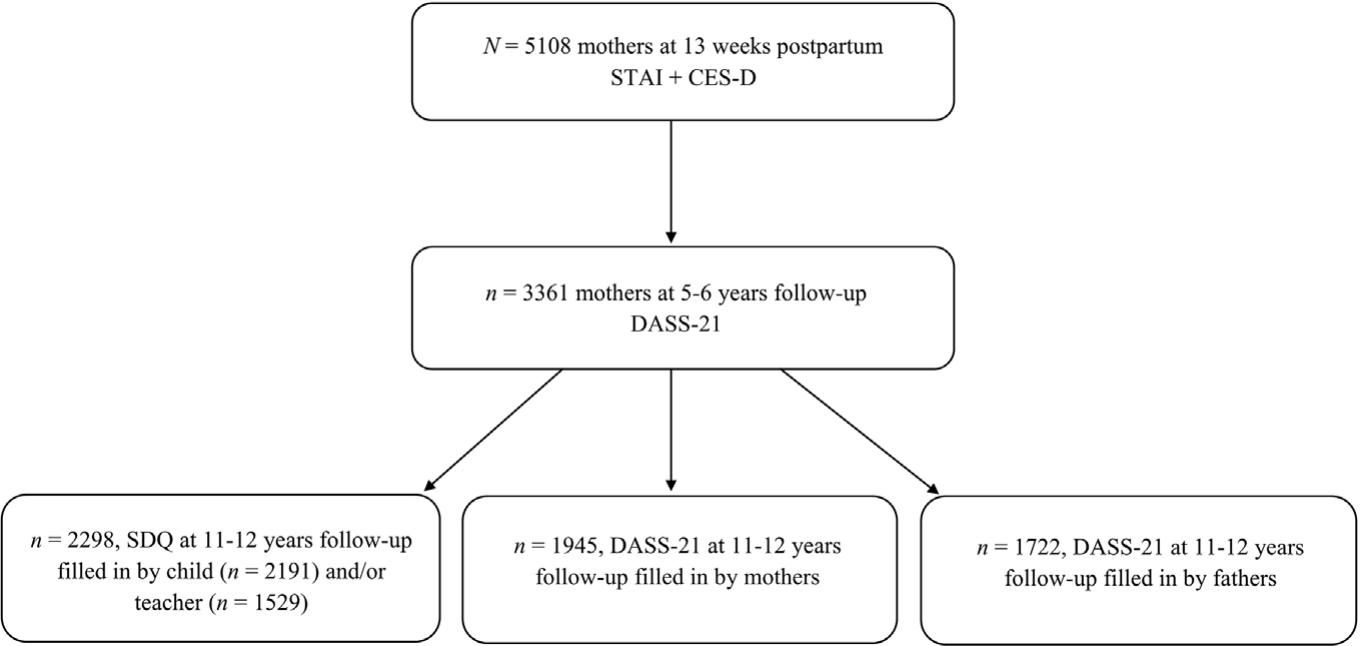
Flowchart of the study population. STAI, State-Trait Anxiety Inventory; CES-D, Center for Epidemiologic Studies Depression Scale; SDQ, Strength and Difficulties Questionnaire; DASS-21, Depression Anxiety Stress Scale.

Between January 2003 and March 2004, 8,266 pregnant women living in Amsterdam were included in the cohort. Of these, 5,108 women reported information on postpartum anxiety and depressive symptoms through questionnaires at 13 weeks after childbirth. For the 5–6 years follow-up measurement, 3,361 biological mothers provided information on anxiety and depressive symptoms. For the 11–12 years follow-up measurement, data on mental health problems from children, their biological mothers, biological fathers, and teachers were collected for up to 2,298 subjects, see flowchart in **Figure 1**.

### Measures

#### Determinants

Maternal postpartum general anxiety was assessed at 13 weeks postpartum using the validated Dutch version of the State-Trait Anxiety Inventory (STAI) (50, 51). The state anxiety subscale contains 20 items, rated on a 4-point scale (1 = rarely or none of the time to 4 = most or all of the time). It measures temporarily experienced anxiety (51), with a higher total score (range 20-80) of the state anxiety subscale indicating more anxiety. In our study, Cronbach’s alpha of the subscale was 0.94.

Maternal postpartum depressive symptoms were assessed using the validated Dutch version of the Center for Epidemiologic Studies Depression Scale (CES-D) (52, 53). The CES-D comprises 20 items on the frequency of depressive symptoms experienced during the past week, rated on a 4-point scale (0 = rarely or none of the time to 3 = most or all of the time). It provides a total score on depressive symptomatology (range 0-60), with higher scores indicating more depressive symptoms. The CES-D corresponds well with clinical measures of depression (53, 54). In the present study, Cronbach’s alpha was 0.90.

Maternal anxiety and depressive symptoms at the 5–6 years follow-up measurement were assessed using the validated Dutch version of the Depression Anxiety Stress Scale (DASS-21) (55, 56). We used two 7-item subscales of the DASS-21 measuring, respectively, anxiety and depression over the last week rated on a 4-point scale (0 = did not apply to me at all, to 3 = applied to me very much or most of the time). For each subscale, sum scores were calculated and then multiplied by two (range 0–42), with higher scores indicating more anxiety or depressive symptoms (55). The DASS-21 has been shown to adequately discriminate between anxiety and depression (57). In the present study, Cronbach’s alphas of the DASS-21 at the 5-6 years follow-up measurement were 0.74 and 0.82 for anxiety and depressive symptoms, respectively.

#### Outcomes

At the 11-12 years follow-up measurement, paternal anxiety and depressive symptoms were assessed using the Dutch version of the Depression Anxiety Stress Scale (DASS-21) (55). Cronbach’s alphas of the DASS-21 were 0.68 and 0.85 (for paternal anxiety and depressive symptoms, respectively.

Emotional problems of the child were assessed using the Dutch version of the Strengths and Difficulties Questionnaire (SDQ) (58, 59), filled out by the child and a teacher of the child. The SDQ contains five subscales with a total of 25 items, rated on a 3-point scale (0 = not true, to 2 = certainly true). For this study, we used the 5-item subscale emotional symptoms, with a higher subscale score indicating a higher risk for emotional problems (range 0-10). The SDQ has good psychometric properties and the subscale emotional symptoms corresponds well with measures of anxiety and depressive symptoms in children (60). In the present study, Cronbach’s alpha was 0.62 for the child-reported subscale and 0.72 for the teacher-reported subscale. These values are in line with alphas reported in previous studies (59, 61).

#### Covariates

Potential confounders were selected *a priori* or based on previous studies investigating the association between maternal affective symptoms in the peripartum and early childhood period and child and parental mental health outcomes (12, 13, 62). Confounders/covariates included demographic variables assessed at 11-12 years follow-up, i.e. parental age (years), educational level (low, moderate, high based on the highest completed school/degree) and employment status (yes, no), and cohabitant status (married/cohabiting, divorced/not living together anymore, other), family financial situation (inadequate, adequate, more than adequate perceived income to live), and maternal ethnicity (Dutch, other-Western, non-Western based on country of birth of the mother) and parity (primi, multi), gestational age (weeks), birth weight (grams), child gender and pubertal stage [assessed by the pubertal development scale (PDS)] and maternal anxiety and depressive symptoms (assessed by the DASS-21). Information on confounders/covariates was obtained *via* the parent-reported questionnaires at 11–12 years follow-up and data of medical registries routinely recorded by midwives and obstetricians [Dutch Perinatal Registration (Perined)] (63, 64).

### Statistical Analysis

First, descriptive statistics were performed to illustrate participant characteristics. We calculated Pearson’s *r* correlation coefficients between measures of maternal anxiety and depressive symptoms postpartum, at child age 5–6 years and concurrent parental symptoms and child emotional problems at 11–12 years follow-up. Further, to examine possible differences of child gender in parental anxiety and depressive symptoms and child emotional problems at 11–12 years follow-up we used independent t-tests. For non-response analysis, independent t-tests and chi-square tests were used for continuous and categorical variables, respectively. Secondly, to analyze the associations between maternal anxiety and depressive symptom scores in the postpartum period at 13 weeks after childbirth and early childhood period at 5–6 years with child emotional problems and paternal anxiety and depressive symptoms at 11–12 years follow-up, multivariable linear regression analyses were conducted. To identify the unique variance contributed by our main predictors, we entered covariates first into the linear regression models; maternal postpartum anxiety or depressive symptoms was entered second, and maternal anxiety or depressive symptoms at age 5–6 years follow-up was added third. As previous research suggests that maternal depressive symptoms in the postpartum and early childhood period are synergistically related to more childhood behavioral and emotional problems (38), a maternal postpartum anxiety (or depressive symptoms) *X* maternal anxiety (or depressive symptoms) at 5–6 years follow-up interaction term was added in a fourth and last step. Thirdly, a second set of multivariable linear regression analyses was used to investigate whether paternal anxiety and depressive symptoms at 11–12 years follow-up moderated the association between maternal anxiety and depressive symptoms postpartum and at 5–6 years follow-up and child emotional problems. Covariates were again entered first into the linear regression models; maternal anxiety or depressive symptoms postpartum and at 5–6 years follow-up was entered second, and paternal anxiety or depressive symptoms at 11–12 years follow-up was entered third. In the fourth and last step, the interaction terms maternal postpartum anxiety *X* paternal anxiety and maternal anxiety at 5–6 years follow-up *X* paternal anxiety were entered. For the depression model, the interaction terms maternal postpartum depression *X* paternal depression and maternal depression at 5–6 years follow-up *X* paternal depression were entered in the fourth and last step.

In line with the recommendations by Dawson (65) for linear regression analyses including interaction terms, the predictor variables and moderators were z-standardized. Self-reported and teacher-reported child emotional problems were combined into one z-standardized outcome using factor analysis (explaining 67.4% shared variance). When only a child self-reported or teacher-reported emotional problem score was available, the respective score was used and z-standardized. So that all outcome variables have the same units, paternal outcomes were also z-standardized. As anxiety/depressive symptoms have been shown to mutually influence each other between parents and between parents and their child (41, 66, 67) and to correct for concurrent symptoms of the other family members, the models for paternal anxiety/depressive symptoms were next to the adjustment for the other confounders and covariates mentioned above, additionally controlled for maternal symptoms and child emotional problems at 11–12 years follow-up, while the models for child emotional problems were adjusted for maternal and paternal anxiety/depressive symptoms at 11–12 years follow-up. The models investigating the contribution of paternal symptoms on the child were only adjusted for concurrent maternal anxiety or depressive symptoms. We examined whether the results may have been biased by collinearity between anxiety or depressive symptoms of the different family members at 11–12 years by inspecting the respective variance inflation factors. Also, to avoid collinearity measures of parental anxiety and depressive symptoms (i.e. the main predictors) were not analyzed in the same regression models, as these measures were strongly correlated, e.g., maternal postpartum anxiety and depression were strongly correlated with *r* = .88, *p* <.001. In addition, as previous research and theory (9, 68) suggest that child gender may moderate the association of parental anxiety and depression with child emotional functioning, we explored whether there were any gender differences in the associations of maternal anxiety or depressive symptoms postpartum or at age 5–6 years and concurrent paternal affective symptoms with child outcome. To do this, we included multiplicative interaction terms between gender and the various parental determinants in the final step of an additional set of stepwise linear regression analyses. Finally, for sensitivity analysis, the main analyses were rerun including married/cohabiting partners only.

Missing data of confounders/covariates and missing data on paternal anxiety and depressive symptoms at 11–12 years when used as confounder were imputed using the multiple imputation procedure. Imputed missing values ranged from 0.1% for gestational age to 27.8% for paternal age in the child emotional problems sample. Test statistics and regression coefficients were averaged across 10 imputed data sets. The level of statistical significance for all analyses was set at α = 0.05. Moreover, we conducted a Bonferroni correction to adjust for multiple comparisons in the link between maternal anxiety (or depressive symptoms) and the three main outcomes (paternal anxiety and depressive symptoms and child emotional problems: *α* level 0.05/3 = 0.017). All statistical analyses were carried out using SPSS version 26.0 for Windows.

### Non-Response Analysis

For non-response analysis, non-responders (those participants who reported data on postpartum maternal anxiety and depressive symptoms but no data on the 5–6 years follow-up or any of the follow-up outcomes, *n* = 2,490) were compared to responders (those participants who reported data on all determinants and for whom at least one outcome variable was available, *n* = 2,618). Compared to responders, non-responding mothers were younger [*M* = 42.9, *SD* = 4.87 vs. *M* = 44.9, *SD* = 4.09; *t*(2647) = -6.351, *p* < .001], more likely to be of non-Western ethnicity [74.4% vs. 52.9%, χ^2^(2)=277.139, *p* < .001] and more likely to be low educated (12.8% vs. 6.6%, χ^2^(2)=26.195, *p* < .001). Children of non-responders had a lower birth weight [*M* = 3436, *SD* = 556 vs. *M* = 3505, *SD* = 532; *t*(5076) = -4.554, *p* < 0.001] and gestational age [*M* = 39.4, *SD* = 1.78 vs. *M* = 39.5, *SD* = 1.67; *t*(5095) = -2.694, *p* = .007]. Furthermore, non-responders reported higher postpartum anxiety [*M* = 36.08, *SD* = 9.83 vs. *M* = 33.97, *SD* = 9.12; *t*(5106) = 7.960, *p* < .001] and depression scores [*M* = 9.66, *SD* = 7.67 vs. *M* = 8.22, *SD* = 6.85; *t*(5106) = 7.094, *p* < .001] compared to responders.

## Results

### Participant Characteristics

Participant characteristics are described in **Table 1**. At the time of the 11–12 years follow-up, mean maternal age was 45.0 (*SD* = 3.96) years. The majority of mothers was of Dutch ethnicity (76.3%), highly educated (73.6%), and married/cohabiting (78.2%). Mean paternal age was 47.1 (*SD* = 5.25) years, and more than half of the fathers were highly educated (56.3%).

**Table 1:** Characteristics of the study sample (*n* = 2298) at the time of the 11–12 years follow-up measurements.

		*M* (*SD*)
*Maternal characteristics*		
Maternal age	Years	45.0 (3.96)
Maternal ethnicity (%)	Dutch	76.3
	Other-Western	12.1
	Non-Western	11.6
Maternal educational level (%)	Low	5.7
	Moderate	14.8
	High	73.6
	Missing	6.0
Maternal employment (%)	Employed	81.9
	Unemployed	13.0
	Missing	5.1
Family financial situation (%)	Inadequate	10.1
	Adequate	56.6
	More than adequate	27.9
	Missing	5.5
Cohabitant status (%)	Married/cohabiting	78.2
	Divorced/not living together anymore	14.4
	Other	2.3
	Missing	5.2
Parity (%)^a^	Primiparous	58.9
	Multiparous	41.1
Postpartum maternal anxiety (%)	Yes	15.4
Postpartum maternal anxiety symptoms	STAI	33.77 (9.10)
Postpartum maternal depression (%)	Yes	12.5
Postpartum maternal depressive symptoms	CES-D	8.06 (6.76)
Maternal anxiety at age 5–6 years (%)	Yes	22.0
Maternal anxiety symptoms age 5–6 years	DASS-21	0.91 (2.98)
Maternal depression at age 5–6 years (%)	Yes	12.7
Maternal depressive symptoms at age 5–6 years	DASS-21	1.88 (3.61)
Maternal anxiety at age 11–12 years (%)	Yes	15.6
Maternal anxiety symptoms at age 11–12 years	DASS-21	1.61 (3.16)
Maternal depression at age 11–12 years (%)	Yes	17.8
Maternal depressive symptoms at age 11–12 years	DASS-21	3.22 (5.00)
*Paternal characteristics*		
Paternal age	Years	47.1 (5.25)
Paternal educational level (%)	Low	5.3
	Moderate	13.1
	High	56.3
	Missing	25.3
Paternal employment (%)	Employed	70.6
	Unemployed	4.8
	Missing	24.5
Paternal anxiety at age 11–12 years (%)	Yes	15.2
Paternal anxiety symptoms at age 11–12 years	DASS-21	1.32 (2.71)
Paternal depression at age 11–12 years (%)	Yes	16.7
Paternal depressive symptoms at age 11–12 years	DASS-21	3.37 (4.84)
*Child characteristics*		
Age child	Years	11.6 (0.32)
Child gender (%)	Boys	49.8
	Girls	50.2
Gestational age	Weeks	39.5 (1.68)
Birth weight	Grams	3515 (535)
Pubertal stage	Score	7.83 (2.81)
Child emotional problems, self-reported	SDQ	1.93 (1.83)
Child emotional problems, teacher reported	SDQ	1.12 (1.62)

M, mean; SD, standard deviation; STAI, State-Trait Anxiety Inventory; CES-D, Center for Epidemiologic Studies Depression Scale; DASS-21, Depression Anxiety Stress Scale. SDQ, Strength and Difficulties Questionnaire.

^a^Parity was assessed at the time of the postpartum measurement.

**Table 2** shows correlations between maternal anxiety and depressive symptoms postpartum and at 5–6 years follow-up, concurrent parental anxiety and depressive symptoms, and child emotional problems at 11–12 years follow-up. Maternal postpartum anxiety was positively and moderately correlated with maternal anxiety at 5–6 years follow-up and positively but weakly with paternal anxiety at 11–12 years. Moreover, maternal postpartum depressive symptoms were positively and moderately correlated with maternal depression at 5–6 years follow-up and positively but weakly with concurrent paternal depressive symptoms. Maternal anxiety and depressive symptoms at all assessment time points and paternal anxiety and depressive symptoms were positively but weakly correlated with child emotional problems at age 11–12 years with one exception: Maternal anxiety at 5–6 years was not significantly correlated with child emotional problems, see **Table 2**. Child gender did not influence the levels of maternal anxiety [boys: *M* = 1.61, *SD* = 3.15 vs. girls: *M* = 1.61, *SD* = 3.17; *t*(1943) = 0.05, *p* = .959] and maternal depression [boys: *M* = 3.35, *SD* = 4.94 vs. girls: *M* = 3.09, *SD* = 4.85; *t*(1943) = 1.17, *p* = .243] or paternal anxiety (boys: *M* = 1.39, *SD* = 2.84 vs. girls: *M* = 1.25, *SD* = 2.58; *t*(1720) = -0.33, *p* = .741] and paternal depression [boys: *M* = 3.33, *SD* = 4.75 vs. girls: *M* = 3.41, *SD* = 4.93; *t*(1720) = 1.08, *p* = .279] at 11–12 years follow-up. Combined self- and teacher reported scores of child emotional problems differed significantly between boys and girls, with boys having lower emotional problem z-scores than girls [boys: *M* = -0.11, *SD* = 0.94 vs. girls: *M* = 0.13, *SD* = 1.04; *t*(2189)=5.68, *p* <.001].

**Table 2:** Bivariate correlations between maternal anxiety and depressive symptoms postpartum and at 5–6 years, maternal and paternal anxiety and depressive symptoms, and child emotional problems at child age 11–12 years.

Variable	Maternal postpartum anxiety	Maternal postpartum depressive symptoms	Maternal anxiety at 5–6 years	Maternal depressive symptoms at 5–6 years	Maternal anxiety at 11–12 years	Maternal depressive symptoms at 11–12 years	Paternal anxiety at 11–12 years	Paternal depressive symptoms at 11–12 years	Child emotional problems at 11–12 years
Maternal postpartum anxiety		.88***	.26***	.30***	.24***	.30***	.10***	.07**	.07***
Maternal postpartum depressive symptoms			.24***	.32***	.25***	.32***	.12***	.08**	.09***
Maternal anxiety at 5–6 years				.47***	.40***	.27***	.14***	.09***	.04
Maternal depressive symptoms at 5–6 years					.26***	.38***	.12***	.08**	.06**
Maternal anxiety at 11–12 years						.49***	.18***	.14***	.12***
Maternal depressive symptoms at 11–12 years							.14***	.17***	.12***
Paternal anxiety at 11–12 years								.51***	.09***
Paternal depressive symptoms at 11–12 years									.10***
Child emotional problems at 11–12 years									

**p < .01, ***p < .001.

### Associations Between Maternal Anxiety and Depressive Symptoms Postpartum and at Child Age 5–6 Years and Child Emotional Problems, Paternal Anxiety, and Paternal Depressive Symptoms at 11–12 Years Follow-Up

**Table 3** presents associations of continuous measures of maternal anxiety and depressive symptoms postpartum and at 5–6 years follow-up with paternal anxiety and depressive symptoms and child emotional problems at 11–12 years follow-up. Associations adjusted for affective symptoms of the other family members and demographic and perinatal/obstetric confounders and covariates at 11–12 years and *R* ^2^ change for each step of the regression analyses are shown.

**Table 3:** Associations of maternal anxiety and depressive symptoms postpartum and during early childhood with z-standardized child emotional problems, paternal anxiety, and paternal depressive symptoms at child age 11–12 years.

	Child emotional problems(*n* = 2298)			Paternal anxiety symptoms(*n* = 1722)			Paternal depressive symptoms(*n* = 1722)		
*Maternal anxiety*	Δ*R* ^2^	B (95% CI)	*p*	Δ*R* ^2^	B (95% CI)	*p*	Δ*R* ^2^	B (95% CI)	*p*
Step 1	.036***			.040***			.060***		
Covariates									
Step 2	.010			.002*			.001		
Postpartum anxiety		0.03 (-0.01; 0.08)	.135		0.05 (0.00; 0.10)	.042		0.31 (-0.02; 0.08)	.235
Step 3	.001			.007***			.003*		
Maternal anxiety at child child age 5-6 years		-0.03 (-0.08; 0.02)	.199		**0.09 (0.04; 0.14)**	**.001**		0.06 (0.01; 0.11)	.024
Step 4	.000			.000			.001		
Postpartum anxiety *X* anxiety at child age 5-6 years		0.02 (-0.01; 0.05)	.244		0.01 (-0.03; 0.06)	.616		0.03 (-0.02; 0.08)	.203
*Total R^2^*	.047			.049			.065		
*Maternal depressive symptoms*									
Step 1	.050***			.039***			.060***		
Covariates									
Step 2	.002*			.004*			.001		
Postpartum depressive symptoms		**0.06 (0.01; 0.10)**	**.017**		**0.07 (0.01; 0.12)**	**.013**		0.04 (-0.02; 0.09)	.188
Step 3	.000			.003*			.001		
Maternal depressive symptoms at child age 5–6 years		0.00 (-0.05; 0.04)	.896		**0.07 (0.01; 0.12)**	**.015**		0.03 (-0.03; 0.08)	.315
Step 4	.001			.000			.000		
Postpartum depression *X* depression at child age 5–6 years		0.02 (-0.02; 0.05)	.332		-0.01 (-0.05; 0.03)	.681		-0.01 (-0.05; 0.03)	.643
*Total R^2^*	.052			.046			.062		

Covariates included parental age, educational level and employment status, cohabitant status, family financial situation, and maternal ethnicity, parity, gestational age, birth weight, child gender, and pubertal stage. Also, models were adjusted for maternal and paternal anxiety or depressive symptoms when including child emotional problems as outcome or adjusted for child emotional problems and maternal anxiety/depressive symptoms, respectively, when including paternal anxiety/depressive symptoms as outcome. All predictors and moderators were z-standardized.

Bold text indicates statistically significant associations after Bonferroni correction.

*p < .05, ***p < .001.

Maternal postpartum anxiety was not significantly associated with child emotional problems and paternal outcomes at 11–12 years. Maternal anxiety at 5–6 years was positively associated with paternal anxiety, but not with paternal depressive symptoms and child emotional problems. Maternal postpartum depressive symptoms were positively associated with child emotional problems and paternal anxiety, but not with paternal depressive symptoms at 11–12 years. Maternal depressive symptoms at 5–6 years were associated with higher levels of paternal anxiety, but not with paternal depressive symptoms and child emotional problems at 11–12 years. The interaction effects of maternal postpartum anxiety *X* maternal anxiety at age 5–6 years and maternal postpartum depression *X* maternal depression at age 5–6 years were not significant for all three outcomes. The interaction effects between gender and maternal anxiety or depressive symptoms postpartum and at 5–6 years on child emotional problems were not significant (postpartum anxiety *B* = 0.04, 95% CI: -0.04; 0.13, *p* = .321 and at 5–6 years *B* = -0.03, 95% CI: -0.12; 0.05, *p* = .463 and postpartum depressive symptoms *B* = 0.06, 95% CI: -0.02; 0.15, *p* = .158 and at 5–6 years *B* = -0.06, 95% CI: -0.15; 0.03, *p* = .179). The explained amount of unique variance of the significant maternal predictors was small according to the benchmarks of Cohen (69).

### Associations Between Paternal Anxiety and Depressive Symptoms at 11–12 Years Follow-Up and Child Emotional Problems and Moderation of Association Between Maternal Symptoms and Child Emotional Problems

Concurrent paternal anxiety at 11–12 years was related with more child emotional problems (*B* = 0.05, 95% CI: 0.00; 0.10 *p* = .041) and explained a significant unique but small amount of variance [*R* ^2^ change = .002, *F*(1,1697), *p* = .041]. The interaction effects of postpartum anxiety *X* paternal anxiety (*B* = -0.01, 95% CI: -0.06; 0.03, *p* = .549) and maternal anxiety at 5–6 years *X* paternal anxiety (*B* = -0.02, 95% CI: -0.06; 0.02, *p* = .227) on child emotional problems were not significant. Concurrent paternal depressive symptoms were also positively associated with more child emotional problems at age 11–12 years (*B* = 0.05, 95% CI: 0.01; 0.10, *p* = .030) and explained a significant unique but small proportion of variance [*R* ^2^ change = .003, *F*(1,1697), *p* = .030]. The interaction effects of Postpartum depression *X* Paternal depression (*B* = -0.02, 95% CI: -0.07; 0.03, *p* = .405) and Depression at 5-6 years *X* Paternal depression (*B* = 0.03, 95% CI: -0.02; 0.07, *p* = .244) on child emotional problems were not significant. Also, the interaction effects between gender and concurrent paternal anxiety or depressive symptoms on child emotional problems were not significant (*B* = -0.02, 95% CI: -0.04; 0.14, *p* = .264 and *B* = -0.08, 95% CI: -0.17; 0.02, *p* = .114, respectively).

### Sensitivity Analyses

Finally, we reran our main analyses among a subsample of married/cohabitating parents only. Overall, we observed a similar pattern of results with some exceptions. First, maternal postpartum depressive symptoms were no longer associated with child emotional problems at age 11–12 years (*B* = 0.05, 95% CI: -0.00; 0.10, *p* = .070). Second, paternal anxiety and depressive symptoms at 11–12 years were not associated with child emotional problems (*B* = 0.05, 95% CI: -0.00; 0.10, *p* = .070 and *B* = 0.05, 95% CI: -0.00; 0.11, *p* = .052, respectively).

## Discussion

In the present large-scale population-based cohort study, maternal anxiety in the early childhood period at child age 5–6 years, but not maternal postpartum anxiety, was specifically but weakly related to more anxiety in fathers when their children were 11–12 years old. While maternal depressive symptoms both postpartum and at child age 5–6 years were positively but weakly related to paternal anxiety at 11–12 years, neither maternal anxiety nor depressive symptoms at both timepoints were related to paternal depressive symptoms at 11–12 years. Maternal postpartum depressive symptoms were associated with more child emotional problems at age 11–12 years. However, maternal anxiety and depressive symptoms postpartum and at child age 5–6 years were not synergistically associated with the child and paternal outcomes. While concurrent paternal anxiety and depressive symptoms at 11–12 years were weakly associated with more child emotional problems, we found no indication of a moderating effect of paternal concurrent affective symptoms on the association between maternal symptoms postpartum and at child age 5–6 years with child emotional problems.

To the best of our knowledge, this study is one of the first to examine the long-term associations of both maternal anxiety as well as depressive symptoms in the postpartum and early childhood period with child and paternal mental health in early adolescence. Regarding fathers, previous studies showed that postpartum paternal and maternal anxiety as well as depression are associated (70, 71). Ierardi et al. (72) found that at 3 months postpartum, higher levels of maternal depression were related to more paternal anxiety, while higher levels of maternal anxiety predicted more paternal anxiety and depression. Our study extends previous work, by indicating that maternal postpartum depressive symptoms and maternal depressive symptoms at child age 5–6 years were associated with more paternal anxiety at child age 11–12 years, even after adjustment for concurrent maternal anxiety and child emotional problems. One might speculate that the experience of depression of the partner in combination with the care for an infant or young child might lead to more worries, hypervigilance, emotional distress and a higher caregiver burden in fathers, possibly leading to persistently increased paternal anxiety (73). We did not find evidence for a synergistic effect of maternal depression postpartum and at 5–6 years on paternal anxiety, but previous studies have shown that women who have experienced postpartum depression subsequently are at high risk for recurrent depressive episodes (37, 38, 74). An alternative possible explanation might be the development or increase of paternal anxiety over time *via* increased relationship distress or shared worries about unemployment or financial problems (41, 75). Interestingly, only maternal anxiety at child age 5–6 years but not in the postpartum period was associated with paternal anxiety. In the postpartum period, challenges raised by parenthood are still new, but the phase in partnership and family life, the parents are situated in when children are aged 5–6 or 11–12 years are more similar and can be characterized by more affective concordance or disturbances in parental functioning and more relationship distress, which, in turn, might play a role in increasing anxiety in fathers (76, 77). However, effect sizes were generally small and maternal anxiety and depressive symptoms in most models were not related to anxiety and depressive symptoms in fathers. This suggests that most fathers seem to be resilient and thus not prone to developing long-term mental health problems due to maternal anxiety and depression of their partners.

Regarding the child, we found that only maternal postpartum depressive symptoms were positively but weakly associated with more child emotional problems at age 11–12 years. This supports the view of the postpartum period as a sensitive period, during which the child is particularly susceptible for the adverse effects of maternal depressive symptoms (78, 79). Potential mechanisms possibly underlying the link between maternal postpartum depressive symptoms and child emotional problems include, next to genetic vulnerability, a lack of maternal sensitivity, inadequate mother-infant interaction and altered infant neurodevelopment possibly due to understimulation (9, 21, 28, 80). Although previous studies suggest that concurrent maternal depression exacerbates the association between maternal postpartum depression and emotional problems of children and adolescents (36–38), we did not observe any evidence that maternal anxiety and depression postpartum and at 5–6 years synergistically increased child emotional problems in early adolescence. The present study had a longitudinal design and adjusted for concurrent affective symptoms of both parents, in contrast to the previous rather small-scale studies by Josefsson and Sydsjo (37) and by Closa-Monasterolo et al. (38), which analyzed maternal depression in childhood as cross-sectional determinant of child outcome. Consistent with prior research (43–45), paternal concurrent affective symptoms were positively related to more child emotional problems in early adolescence. However, paternal concurrent anxiety or depressive symptoms did not moderate the association between maternal symptoms and child emotional problems. Possibly, other moderating factors including involvement and quality of the father-child relationship, paternal sensitive parenting, or the extent of impaired parenting due to psychopathology of both parents play a more important role in the relation between maternal affective symptoms and child emotional development (9, 22, 46, 47, 81). Also, regarding gender interactions, no significant effects were found. Previous research into the moderating effect of gender on the association between parental affective symptoms and children’s emotional functioning has shown inconsistent results (9, 68, 82). Sensitivity analysis showed that, when only married or cohabiting parents at 11–12 years were included, maternal postpartum depression was no longer associated with child emotional problems, as were paternal concurrent anxiety and depressive symptoms with child emotional problems. This might indicate that parental relationship conflict and family functioning possibly mediate the relation of maternal postpartum depression with the development of child emotional problems (41, 83). Finally, with respect to the long-term association of maternal postpartum anxiety with child development, to date, there is only little research available reporting conflicting results, and two reviews concluded that more evidence is needed (15, 16). In our sample, maternal anxiety was not related to child emotional problems. In contrast to previous studies using parent reported information (37, 38), we assessed child emotional problems by use of self-report by children combined with teacher report. This improves the validity of our results and might also partly account for the small effect sizes observed in the present study, as parent report of depressed parents on their children’s mental health problems has been shown to be potentially biased (84, 85).

One of the strengths of this large-scale prospective cohort study is the information on a large number of potential confounders and covariates. Moreover, we were able to control for concurrent affective symptoms of the other family members. One might argue that we adjusted the regression models overly conservatively, as some of the confounders and covariates have also been identified as predictors of anxiety and depressive symptoms (86). Therefore, we cannot completely rule out that we underestimated effect sizes. However, our approach is in line with previous longitudinal studies (13, 87) and supports the robustness of our findings. Also, we did not observe multicollinearity between concurrent affective symptoms of the family members.

The study also had several limitations. First, the long follow-up period has led to attrition and we cannot eliminate the risk that selection bias affected our results. Compared to non-responders, responding mothers were more often Dutch, highly educated and had lower postpartum anxiety and depressive symptom scores, which also might have led to a conservative estimation of associations. Second, postpartum anxiety and depressive symptoms were assessed by the STAI and the CES-D, respectively, while anxiety and depressive symptoms at 5–6 years and at 11–12 years were assessed using the DASS-21. However, all three instruments have been demonstrated to be good predictors of clinical anxiety and depression, respectively, and the STAI and CES-D correlate moderately-to-strongly with the DASS-21 anxiety or depression subscale, respectively (88–91). Third, we only had information about maternal anxiety and depressive symptoms at the three follow-up time points. Moreover, as fathers were not included in the present study before the 11–12 years follow-up measurement, associations with child emotional problems could only be analyzed in a cross-sectional manner. Preferably, in future longitudinal studies affective symptoms in both parents should be assessed more often, to obtain more information on their course over time. Furthermore, children should preferentially be followed until late adolescence, as the prevalence of affective symptoms increases during adolescence (92–94). Fourth, although we adjusted for several shared environmental confounders and covariates, the study design did not allow accounting for familial confounding such as the contribution of shared genetic risk factors between parents and offspring. Lastly, we investigated the possible specific and synergistic impact of maternal mental health factors in the postpartum and early childhood period on several child and paternal affective outcomes in early adolescence. Although the various tests do not necessarily constitute independent hypotheses, we adjusted for multiple testing by applying a Bonferroni correction, which did not lead to a change of the interpretation of our main results. Nevertheless, one should bear in mind that for those associations revealed to be statistically significant we only observed small effect sizes indicating that our findings are of little clinical significance.

Our results illustrate that fathers seem to be affected only to a small extent by maternal postpartum anxiety or depression. Future work should gain more insight into resilience factors in fathers. By this, preventive interventions and treatment for maternal postpartum anxiety and depression could be improved and developed by father participation. Finally, research is needed elucidating mechanisms involved in the long-term association between maternal postpartum anxiety and depressive symptoms and child emotional problems in early adolescence.

## Conclusion

This population-based cohort study aimed to increase our understanding of the long-term impact of maternal anxiety and depression in the postpartum as well as early childhood period on the father and the child. Maternal postpartum depressive symptoms, maternal depressive symptoms at child age 5–6 years, and maternal anxiety at 5–6 years were positively but weakly related to paternal anxiety at child age 11–12 years. Notably, only maternal postpartum depression was, also weakly, associated with more child emotional problems at age 11-12 years, suggesting that other factors contribute more significantly to child emotional problems in early adolescence.

## Data Availability Statement

The datasets presented in this article are not readily available because the data are not publicly available and were used under license for the current study. Requests to access the datasets should be directed to the PI of the ABCD study: TV, t.vrijkotte@amsterdamumc.nl.

## Ethics Statement

The studies involving human participants were reviewed and approved by the Central Committee on Research Involving Human Subjects in The Netherlands and the medical ethics research committee of the Amsterdam Medical Center (METc AMC), Amsterdam, The Netherlands. Written informed consent to participate in this study was provided by all adult participants and for the participating children by their legal guardian/next of kin.

## Author Contributions

The study was designed by ALW, TV, and JH. SR and TV provided the study data. ALW, PP, and JH analyzed the data and drafted the manuscript. All authors contributed to the article and approved the submitted version.

## Funding

This part of the ABCD-study was financially supported by the Netherlands Organization for Health Research and Development (grants 21000076, 92003489, and 040-00812-98-11010), Dutch Heart Foundation (grant 2007B103), and Sarphati Amsterdam.

## Conflict of Interest

The authors declare that the research was conducted in the absence of any commercial or financial relationships that could be construed as a potential conflict of interest.
